# Editorial: Understanding the materno-fetal interface during microbial infections, Volume II

**DOI:** 10.3389/fmicb.2022.1072491

**Published:** 2022-10-28

**Authors:** Demba Sarr, Ricardo E. Fretes, Ulrike Kemmerling, Sodiomon B. Sirima

**Affiliations:** ^1^Department of Infectious Diseases, University of Georgia, Athens, GA, United States; ^2^Histology and Embryology Department, School of Medicine, Universidad Nacional de Córdoba and Villa María, Córdoba, Argentina; ^3^Instituto de Ciencias Biomédicas, Facultad de Medicina, Universidad de Chile, Santiago, Chile; ^4^Health Action Research Group, Ouagadougou, Burkina Faso

**Keywords:** maternal-fetal interface, placenta, decidua, infection, immunity, pregnancy

The placenta and the decidua represent the maternal-fetal interface. How the different components of the maternal-fetal interface orchestrate key immunological responses and maintain homeostasis during pregnancy to sustain maternal tolerance and prevent infections are important research questions. Our understanding of these key responses is rapidly growing. However, our knowledge of how the maternal-fetal interface responds and specifically eliminates pathogens and how these pathogens can overcome these responses remain limited.

The placenta is composed of different trophoblast populations, such as the extravillous trophoblast relevant during implantation and the villous trophoblast, that form part of the placental anatomical barrier. The villous trophoblast is formed by cytotrophoblast cells that fuse and form the multinucleated syncytiotrophoblast that presents innate immune functions, participating in the prevention of infection and maintenance of pregnancy.

Using a pregnant mouse model of sublethal prenatal endotoxaemia to mimic bacterial infection during pregnancy, Reginatto et al. demonstrated an increased risk of fetal death or early labor. Further data analysis performed on tissues from fetuses that did not show signs of death and from dams that did not undergo early labor indicated a decrease in placental weight associated with maternal and placental pro-inflammatory responses and alteration of maternal and placental lipid homeostasis throughout pregnancy. These findings support and expand the knowledge on the negative impacts of bacterial infection during pregnancy, but further investigations are needed to clarify the mechanisms that lead to altered expression of lipid transporters. As part of the maternal-fetal interface, the placenta is involved in fetal development maintains homeostasis, and fulfills its immunological role. However, pathogens such as *Toxoplasma gondii, herpes simplex virus, cytomegalovirus, Listeria monocytogenesis*, and *Plasmodium falciparum* can break this barrier with negative consequences to the developing fetus.

Using an *in vitro* model system of the human villous trophoblast cells, Johnson et al. demonstrated that these cells are also gatekeepers that limit *L. monocytogenes* infection. The bacteria induce an inflammatory response of the cytotrophoblast and syncytiotrophoblast with a signature gene associated with poor pregnancy outcomes. The authors concluded that the initial response of *L. monocytogenes*-infected trophoblast cells might have beneficial effects by recruiting innate immune cells and activating the inflammasome. However, if the pathogen is not cleared, a sustained and uncontrolled response may favor poor pregnancy outcomes of listeriosis.

Maternal microbiota affects fetal development. It may cause adverse pregnancy outcomes, which can negatively impact the health of the offspring. Using a critical and conclusive review of the Research Topic through Web of Science and Pubmed, Yao et al. concluded that the maternal microbiome is closely associated with the health of the fetus. They indicated that the maternal diet can affect the offspring's gut microbes through lactation, suggesting breastfeeding moms should diet carefully for themselves and their newborn babies. Moreover, the maternal microbiota is an essential element that regulates the fetus's innate immunity during pregnancy. During birth and after, the infant microbiota will continue to develop and train innate and adaptive immunity, promoting a healthy or normal immune system. Therefore, alteration or disorder of maternal microbiota during pregnancy can cause adverse pregnancy outcomes and vertical transmission of the maternal microbiome.

Infection with *Toxoplasma gondii*, an obligate intracellular parasite, can cause poor pregnancy outcomes or congenital toxoplasmosis. *T. gondii* infection during pregnancy leads to an inflammatory response at the maternal-fetal interface making the pregnancy difficult to maintain. Toll-Like Receptors (TLRs) are pathogen recognition receptors expressed by innate immune cells. These TLRs allow innate immune cells to easily detect pathogen-associated molecular patterns and recognize them for initiating immune responses. Using *Tlr2*-deficient mice, Ikeda et al. demonstrated that TLR2 might have a role in developing toxoplasmosis during pregnancy. *T. gondii*-infected *Tlr2*-deficient mice have significantly lower serum IFN levels than control mice at late pregnancy, suggesting that regulation of TLR2 signaling may be a potential target for controlling congenital toxoplasmosis.

A review article by Rojas-Pirela et al. summarized important insights into our understanding of the role of the mammalian placenta in congenital transmission by apicomplexan parasites, including *Plasmodium, Babesia, Toxoplasma*, and *Neospora* species. *Plasmodium falciparum, Babesia, Toxoplasma gondii*, and *Neospora caninum* adhere to epithelial or endothelial cells inside the placenta, posing significant risks to pregnancy in both humans and animals. The parasites break through the placental barrier and cause congenital infection. The authors concluded that these apicomplexan parasites use similar adhesion mechanisms to the placenta. Understanding the molecular interactions between host genes/proteins and parasite genes/proteins is essential for preventing maternal and congenital infections.

Chua et al. reviewed evidence from human and mouse studies to highlight the potential mechanisms underlying placental pathologies in malaria during pregnancy. Mainly, the dysregulated placental vascularization leading to irreversible damage, abnormal placental structure and apoptosis, the role of cytokines/chemokines, placental hormones, and Complement and coagulation cascades were analyzed. The authors indicated that more studies are needed to determine whether beneficial therapies in mice can be achieved in humans. Furthermore, from dysregulated autophagy and heat shock proteins in rescue mechanisms to the limited efficacy or the absence of a vaccine for pregnant women, the continuous use of *in vitro* cell culture model systems mimicking the placenta and *in vivo* models to delineate pathways in this organ during malaria, and the fact that malaria during pregnancy has remained a public health issue and a huge threat to both the future mother and her developing fetus, indicate that more research is still needed.

Fakonti et al. reviewed the role of Hofbauer cells, placental villous macrophages of fetal origin, in response to infection during pregnancy. Understanding these cells' role is still rudimentary, but evidence suggests that they are essential for placental development and homeostasis. The authors used Pubmed and Scopus Web searches to identify 86 studies for their review. They found that a variety of pathogens can infect Hofbauer cells, and their hyperplasia is a common feature. Although these cells can replicate and transmit pathogens to other cells, they can also eliminate pathogens by a variety of mechanisms, including phagocytosis, cytokine-mediated pathogen elimination, release of macrophage-extracellular trap, and Hofbauer cell-antibody-mediated neutralization. Additional investigations are needed for a broader understanding of the role of these cells at the maternal-fetal interface.

In conclusion, we still need more investigations to understand better the role of the maternal-fetal interface during bacterial, viral, and parasitic infections. Deciphering the molecular interactions between host genes/proteins and pathogen genes/proteins and the impact of infection on homeostasis will facilitate the discovery of new therapy and preventive strategies in maternal and/or vertical transmission during pregnancy. More multidisciplinary, comprehensive, larger-scale studies will be required to confirm fragmentary or inconsistent findings and bring new insights into our understanding of the complex mechanisms occurring at the maternal-fetal interface. Evaluating the role of the microbiome (maternal and fetal) is also crucial.

## Author contributions

DS drafted the Editorial. However, all authors listed have made a substantial, direct, and intellectual contribution to the Editorial and approved it for publication.

## Conflict of interest

The authors declare that the research was conducted in the absence of any commercial or financial relationships that could be construed as a potential conflict of interest.

## Publisher's note

All claims expressed in this article are solely those of the authors and do not necessarily represent those of their affiliated organizations, or those of the publisher, the editors and the reviewers. Any product that may be evaluated in this article, or claim that may be made by its manufacturer, is not guaranteed or endorsed by the publisher.

